# Correlates of gallbladder stones among patients with sickle cell disease: A meta‐analysis

**DOI:** 10.1002/jgh3.12622

**Published:** 2021-08-07

**Authors:** Sagad O O Mohamed, Omer A O Ibrahim, Dahlia A A Mohammad, Almigdad H M Ali

**Affiliations:** ^1^ Department of Paediatrics and Child Health, Faculty of Medicine University of Khartoum Khartoum Sudan; ^2^ Department of Internal Medicine University of Khartoum Khartoum Sudan; ^3^ Department of Surgery University of Khartoum Khartoum Sudan

**Keywords:** cholelithiasis, meta‐analysis, prevalence studies, sickle cell disease

## Abstract

Sickle cell disorders are the most common hemoglobinopathies worldwide. Clinical variability of sickle cell disease (SCD) and susceptibility to its complications have been attributed to hematologic, genetic, and other influencing factors. This review aimed to provide further summary and analyses of the prevalence and factors associated with cholelithiasis among patients with SCD. A systematic database search was conducted in MEDLINE (PubMed), ScienceDirect, Google Scholar, World Health Organization Virtual Health Library, Cochrane Library databases, and System for Information on Gray Literature in Europe (SIGLE). Pooled prevalence, odds ratio (OR), and standardized mean difference (SMD) with the corresponding 95% confidence interval (CI) were calculated using Comprehensive Meta‐Analysis Software version 3.3. A total of 34 studies that fulfilled the eligibility criteria were included in the analyses. The overall prevalence of cholelithiasis among SCD patients was 25.3% (95% CI 19.4–32.3%). The risk of developing cholelithiasis was significantly associated with lower total hemoglobin level (SMD = −0.45; *P* = 0.002), lower hemoglobin F (HbF) level (SMD = −0.85; *P* = 0.003), higher total serum bilirubin level (SMD = 1.15; *P* < 0.001), higher reticulocytes count (SMD = 0.44; *P* = 0.007), and UDP‐glucuronosyltransferase‐1A1 enzyme (UGT1A1) promoter polymorphism. This review provides a comprehensive view of the high rate of cholelithiasis and its associated factors in SCD patients.

## Graphical abstract

This meta‐analysis showed that up to a quarter of all sickle cell disease patients are susceptible to cholelithiasis. The risk of developing cholelithiasis is significantly associated with older age, lower total hemoglobin level, lower hemoglobin F level, higher total serum bilirubin level, higher reticulocytes count, and UDP‐glucuronosyltransferase‐1A1 enzyme (UGT1A1) promoter polymorphism.

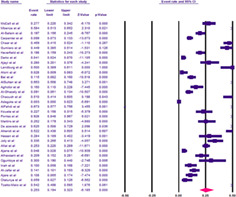

## Background

Sickle cell disorders are the commonest major hemoglobinopathies worldwide and they are mainly distributed in the regions of sub‐Saharan Africa, Mediterranean, Middle East, and the Indian subcontinent.^1^, ^2^, ^3^ Sickle cell disease (SCD) is caused by a point mutation in the B‐globin gene, which results in abnormal reversible sickling of RBCs when they get deoxygenated. Recurrent sickling episodes will result in permanently sickled RBCs with disrupted cell membranes, leading to a cascade of pathophysiological consequences.^4^, ^5^, ^6^, ^7^

SCD is associated with protean clinical presentation of hemolytic anemia, painful crisis, aplastic crisis, and sequestration crisis.^5^, ^6^, ^7^ Hepatobiliary abnormalities are common in SCD and they are mainly attributed to the chronic hemolytic nature of the disease and the resultant increase in unconjugated bilirubin, favoring the formation of gallbladder stones.^8^, ^9^ Individuals with chronic hemolysis, including SCD, have an increased risk of cholelithiasis with pigmented stones that result from increased unconjugated bilirubin excretion due to catabolic breakdown of heme.^10^, ^11^ The bilirubin precipitation resulting from chronic hemolysis is a determinant factor for the formation of gallstones in SCD.^10^, ^11^

While most of these gallbladder stones are asymptomatic, some of them can result in infection and inflammation of the gallbladder, along with other surgical complications in the biliary tract.^8^, ^9^ Cholelithiasis can lead to cholecystitis acute and chronic cholecystitis, cholangitis, common bile duct (CBD) obstruction, and gallstone pancreatitis.^12^, ^13^, ^14^

It has been reported that cholelithiasis and its complications are more common and responsible for higher levels of morbidity in SCD patients. For example, CBD obstruction has been reported among near half of the symptomatic children and young adults with SCD, which is higher than the rates reported among the general population who underwent cholecystectomy (10–15%).^10^, ^12^, ^13^ A previous analysis of 149 415 patients with gallbladder diseases from the United States showed that SCD patients had higher complication rates and longer lengths of stay than the general population and these differences are pronounced among those who underwent surgery in the acute setting.^14^

The preponderance of cholelithiasis in SCD has made cholecystectomy the most commonly performed surgical procedure in SCD patients, comprising up to 40% of the surgical procedures.^11^, ^12^, ^13^, ^14^, ^15^ Moreover, prophylactic cholecystectomy has been widely described and considered for SCD patients with asymptomatic cholelithiasis because of several reasons such as the noted higher prevalence of cholelithiasis in SCD patients and the similarity between clinical symptoms of biliary complications and vaso‐occlusive crisis (i.e. acute abdominal pain, jaundice, fever, and leukocytosis), which may render definitive diagnosis and management difficulty.^12^, ^16^, ^17^ However, elective cholecystectomy for SCD patients remains a matter of debate.^11^, ^18^

Previous reviews showed that clinical variability of SCD and susceptibility to its complications, such as cerebrovascular accidents and sickle nephropathy, are attributed to several hematological and clinical factors.^19^, ^20^, ^21^ Likewise, we hypothesized that the manifestation of cholelithiasis in SCD patients has contributing factors responsible for modulating the clinical behavior of the disease. Cholecystectomy is not risk‐free, and cholelithiasis has been described to be more common in SCD patients and rates of its complications are higher. However, the true prevalence of cholelithiasis in SCD patients remains unknown and understanding of its risk factors is still limited. In this review, we aimed to provide a further summary and overview of the studies that have assessed the frequency and the risk factors of cholelithiasis in SCD patients.

## Methods

### Search strategy and inclusion criteria

This review's protocol was registered in PROSPERO under the number: CRD 42020219655. The methodology was established based on the Preferred Reporting Items for Systematic Reviews and Meta‐Analyses (PRISMA) statement.^22^ We performed a systematic literature search using the electronic databases of MEDLINE (PubMed), Google Scholar, ScienceDirect, WHO Virtual health library, Cochrane Library databases, and System for Information on Gray Literature in Europe (SIGLE) without limitations regarding sex, race, geographical area, or publication date. The search terms used were “gallbladder,” “gallbladder stones,” “gallstones,” “cholelithiasis,” “cholecystitis,” “cholecystectomy,” and “sickle cell.” Moreover, we manually searched for more studies by screening the references of the included studies in this review.

The retrieved publications were imported into Rayyan software (QCRI, Doha, Qatar; http://rayyan.qcri.org) to expedite initial titles/abstracts screening and duplicates deletion.^23^ The criteria for articles inclusion were cross‐sectional, case–control, or cohort studies that reported specific data on number of SCD patients with and without cholelithiasis. Also, we included studies that reported specific data on the association between cholelithiasis and any background, hematological or genetic risk factors among SCD patients if present. The reported risk factors were included in the meta‐analysis to determine the significance and direction of the association. We excluded case reports, editorials, reviews, abstracts, and studies without sufficient data of interest. All titles and abstracts of articles retrieved from the search were screened for possible inclusion in this review. Then, the full texts of studies deemed to be relevant were reviewed for inclusion according to the defined eligibility criteria.

### Quality assessment and data extraction

Quality assessment of the included studies was done using the Newcastle–Ottawa scale, a tool that determines the quality based on the selection of the study group, comparability of groups, and ascertainment of the exposure and outcomes. Data were extracted by three independent reviewers and any disparity among the reviewers was resolved by discussion and consensus. For qualitative and quantitative data syntheses, we extracted the relevant information from each article and recorded them in a Microsoft Excel spreadsheet.

### Statistical analysis

The statistical analyses were carried out by using Comprehensive Meta‐Analysis Software version 3.3 (Biostat, Engle‐wood, NJ, USA; http://www.Meta-Analysis.com). The heterogeneity was assessed through *I* ^2^ test, which describes the percentage of variability in the effect estimates. In cases where high heterogeneity was detected, we calculated the pooled summary prevalence and odds ratio (OR) from the random‐effects models. We conducted a meta‐regression analysis to determine the extent to which the continuous variables moderated the overall results. Publication bias was determined through Begg's test, Egger's test, and visual examination of the funnel plot.^24^, ^25^ If a publication bias was found, the Duval and Tweedie trim and fill method was used to add possible missing studies and to calculate the adjusted pooled value.^26^

## Results

**Figure 1:**
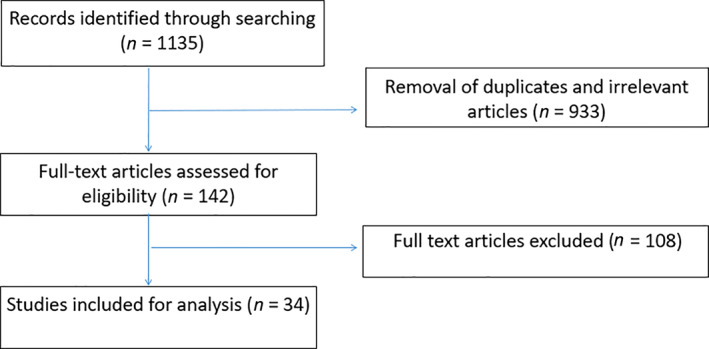
The flow diagram for the process of study selection.

The schematic flow of the study identification and selection process is presented in Figure 1. Initially, the literature search retrieved 1135 published articles. After exclusion of studies because of irrelevance and insufficient data to estimate the outcomes of interest, a total of 34 eligible studies published from 1977 to 2020, which met the eligibility for data extraction and analyses, were used for qualitative and quantitative synthesis; 15 studies from Africa,^27^, ^28^, ^29^, ^30^, ^31^, ^32^, ^33^, ^34^, ^35^, ^36^, ^37^, ^38^, ^39^, ^40^, ^41^ seven studies from the South America and North America,^42^, ^43^, ^44^, ^45^, ^46^, ^47^, ^48^ seven studies from Asia,^8^, ^49^, ^50^, ^51^, ^52^, ^53^, ^54^ and four studies from Europe.^55^, ^56^, ^57^, ^58^ Overall, this analysis included a total of 6771 SCD patients. The main characteristics of the included studies are shown in Table 1.

**Table 1:** Descriptive summary of the studies included in the review

Study	Year	Country	Age group	Number of patients with SCD	Number of patients with cholelithiasis	Factors found to be associated with cholelithiasis
McCall et al.	1977	Jamaica	All	206	57	Retics count, HbF level, serum bilirubin
Mbenza et al.	1994	Congo	All	190	111	Age, leukocyte count
Al‐Salem et al.	1995	Saudi Arabia	Children	305	60	Age, Serum bilirubin
Carpenter et al.	1997	USA	Children	393	39	UGT1A1 promoter polymorphism
Chaar et al.	2002	France	All	324	152	UGT1A1 promoter polymorphism, serum bilirubin
Gumiero et al.	2003	Brazil	Children	225	101	Hb genotypes
Haverfield et al.	2005	Jamaica	All	339	67	UGT1A1 promoter polymorphism
Darko et al.	2005	Ghana	Children	315	13	Sex
Ajayi et al.	2006	Nigeria	Adults	100	28	BMI, Sickle cell crises, Serum bilirubin, ALT, AST
Landburg et al.	2007	Netherland	Adults	76	38	Plasma ADMA concentration
Aloni et al.	2007	DR Congo	Children	108	3	None
Bai et al.	2009	Sudan	Children	261	30	None
AlSultan et al.	2010	Saudi Arabia	All	104	69	Serum bilirubin
Agholor et al.	2011	Nigeria	All	150	24	Age, Hb genotype
Al‐Ghazaly et al.	2011	Yemen	All	252	23	None
Chaouch et al.	2012	Tunisia	Children	102	52	UGT1A1 promoter polymorphism, serum bilirubin
Adegoke et al.	2013	Nigeria	Children	300	1	None
AlFahdi et al.	2013	Kuwait	Adults	104	70	UGT1A1 promoter polymorphism
Koueta et al.	2013	Burkina Faso	Children	110	25	Age, VOC, History of blood transfusion
Pontes et al.	2013	Brazil	All	83	77	Sex, absence of alpha thalassemia (decrease risk), not taking hydroxyurea
Martins et al.	2014	Brazil	All	107	27	Age
De azevedo et al.	2014	Brazil	Adults	72	45	None
Alkendi et al.	2015	Oman	All	136	71	None
Hassan et al.	2015	Nigeria	Adults	67	19	Age, gallbladder volume
Joly et al.	2016	France	Children	158	53	UGT1A1 promoter polymorphism, absence of Alpha globin status, serum bilirubin, Hb level, Hb F, not taking Hydroxyurea, reticulocyte count, VOC, ACS
Allai et al.	2017	France	Children	616	156	ALT, serum bilirubin
Ajane et al.	2017	Nigeria	Children	294	14	Age
Alhawsani et al.	2017	Saudi Arabia	Children	153	32	MCV, Hb SS level
Oguntoye et al.	2017	Nigeria	Adults	50	15	None
Inah et al.	2018	Nigeria	All	120	12	Age, sex, weight, and gallbladder volume
Al‐Jafar et al.	2019	Kuwait	All	220	31	Sex
Ajare et al.	2019	Nigeria	All	130	14	None
Olatunya et al.	2019	Nigeria	All	101	6	UGT1A1 promoter polymorphism, serum bilirubin, Hb F
Tzatto‐Maio et al.	2020	Brazil, Senegal, and France	All	500	271	Age, sex, 20 SNPs in genes encoding immune molecules [Toll‐like receptors (TLR), NK cell receptors (NKG), histocompatibility leukocyte antigens (HLA), MHC class I polypeptide‐related sequence A (MICA), and cytotoxic T‐lymphocyte‐associated antigen 4 (CTLA‐4)]

ACS, acute chest syndrome; ADMA, asymmetric dimethylarginine; ALT, alanine aminotransferase; AST, aspartate aminotransferase; BMI, body mass index; MCV, mean corpuscular volume; SCD, sickle cell disease; UGT1A1: UDP‐glucuronosyltransferase 1A1 enzyme; VOC, vaso‐occlusive crisis.

**Figure 2:**
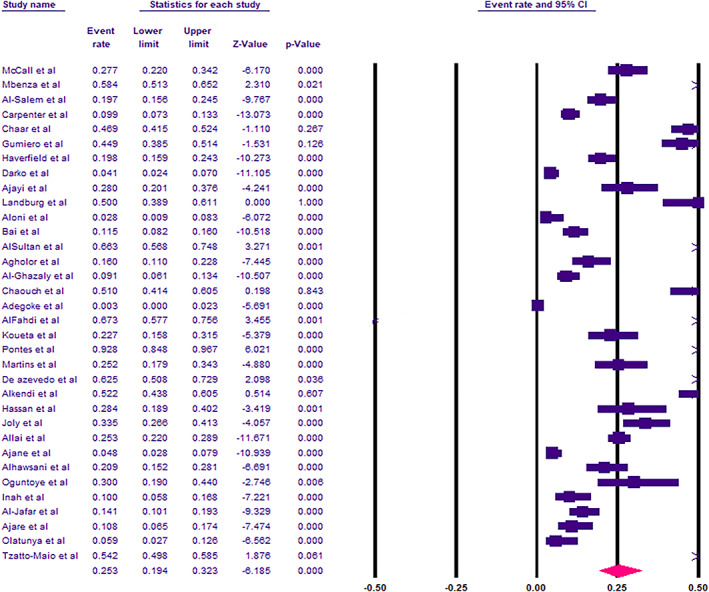
Pooled prevalence of cholelithiasis among patients with sickle cell disease.

The meta‐analysis for the included studies showed that the overall prevalence of cholelithiasis among SCD patients was 25.3% (95% confidence interval [CI] 19.4–32.3%) (Fig. 2). Duval and Tweedie trim and fill method showed that no potential studies are missing, and the adjusted estimate was still significant and similar to the original findings. Meta‐regression analysis showed that the study year did not affect the heterogeneity among studies and had no moderating effects on the prevalence of cholelithiasis (*P* = 0.679). In subgroup analysis based on age groups, the pooled prevalence was higher among adults with SCD (44.1%), where it was 14.9% among children with SCD. There was a significant difference in prevalence between different age groups (*X* ^2^ = 200.4, *P* < 0.001) (Table 2).

**Table 2:** Meta‐analyses of factors associated with cholelithiasis in SCD patients

Variable	Number of included studies	Number of patients	Estimate	Pooled estimate (95% confidence interval [CI])	*P* value
Age group (adults)	6	469	Prevalence	44.1% (95% CI, 29.7–59.6)	<0.001
Total serum bilirubin	9	1640	SMD	1.15 (95% CI, 0.74–1.55)	<0.001
Mean Hb level	7	1415	SMD	−0.45 (95% CI, −0.73 to −0.32)	0.002
Mean HbF %	5	883	SMD	−0.85 (95% CI, −1.38 to −0.27)	0.003
Reticulocytes %	5	773	SMD	0.44 (95% CI, 0.12–0.75)	0.007
HB SS	3	485	OR	1.81 (95% CI, 1.15–2.86)	0.011

OR, odds ratio; SMD, standardized mean difference.

Our review identified several potential risk factors for developing gallstones among SCD patients. However, the meta‐analyses showed that occurrence of gallstones was significantly higher among patients with lower total Hb level (SMD = −0.45; 95% CI, −0.73 to −0.32), lower Hb F level (SMD = −0.85; 95% CI, −1.38 to −0.27), higher total serum bilirubin level (SMD = 1.15; 95% CI, 0.74–1.55), and higher reticulocytes count (SMD = 0.44; 95% CI, 0.12–0.75) (Table 2).

In addition, a number of genetic factors have been assessed by several studies (Table 2). Of these factors, UDP‐glucuronosyltransferase 1A1 enzyme (UGT 1A1) polymorphism was the most common factor linked to higher risk of cholelithiasis in SCD patients, especially the homozygosity for seven thymine‐adenine (TA) repeats. One study by Landburg et al. quantified the relationship between rates of cholelithiasis in SCD and plasma concentration of asymmetric dimethylarginine (ADMA), an endogenous nitric oxide synthase inhibitor.^55^

## Discussion

During the SCD course, the nature of chronic inflammation and hemolysis with increased excretion of bilirubin render SCD patients prone to several complications including the hepatobiliary disorders. Existing evidence from this quantitative meta‐analysis suggest that up to a quarter of all SCD patients are susceptible to cholelithiasis. Moreover, this meta‐analysis showed a remarkably higher prevalence of gallstones in the adults with SCD (44.1%) than that of the adults from general population as reported by previous reviews (10–15%).^59^, ^60^ In addition, we found that the rate of cholelithiasis is higher among adults and those with Hb SS than others. A previous review reported that the rates of gallstones are higher in patients with Hb SS compared with other genotypes such as Hb SC and Hb S‐beta thalassemia.^12^

It is notable that all the identified associated factors with cholelithiasis in this review are correlates of excessive hemolysis. Indeed, gallstones in SCD, as well as other chronic hemolytic disorders, are usually of the black rather than brown pigment type as a result of elevated bilirubin excretion.^12^, ^61^ Gallstones are being recognized with increasing frequency in children with diverse group of etiologies.^62^ Bouge et al. in a six‐year study found that 25% of all gallbladder stones cases in children were due to chronic hemolytic anemia, mainly SCD.^62^

The most frequent genetic factor linked to cholelithiasis in SCD was UGT1A1 enzyme, which is responsible for bilirubin glucuronidation into a water‐soluble form to be excreted in bile.^47^, ^51^ The TATA box promoter region of the gene contains a group of TA repeats, with four alleles described in humans.^58^ These alleles differ in the number of repeats, from five to eight.^58^ Homozygosity of the (TA)7 allele has been described and linked to the unusually high unconjugated bilirubinemia observed in Gilbert's syndrome and various forms of hemolytic disorders such as SCD, beta thalassemia, and hereditary spherocytosis.^58^ Seven of the reviewed studies assessed UGT1A1 polymorphism in SCD and found that the frequencies of (TA)7 and (TA)8 were significantly higher in SCD patients with cholelithiasis. The most significant values were obtained for those who were homozygous for (TA)7 allele or heterozygous (TA)7/(TA)8.^51^, ^58^

There are several implications to our findings. From a clinical perspective, the SCD patients who have one or more of the identified factors in this review are at higher risk of gallstones and should be prioritized for elective cholecystectomy if the practice was adopted and should be included in any screening program for gallstones if present.^63^, ^64^ In general, cholelithiasis and/or its complications should be anticipated in all SCD patients presenting with acute abdominal pain, especially if they are of older age. From a research perspective, the paucity of data on outcomes of cholelithiasis and associated factors needs to be addressed and robust clinical prediction models need to be developed for better management.

A limitation to be considered in this review is the inclusion of studies published only in English, which could compromise representativeness. Another limitation is the paucity of publications on the outcomes of cholelithiasis. There is a need for large prospective studies with appropriate controls to elaborate more on these issues.

## Conclusion

This review provides a comprehensive view of the high rate of cholelithiasis and its associated factors in SCD patients. Most of the associations yielded from our analyses were proxies of excessive hemolysis. A more inclusive consideration of the existing evidence will raise healthcare providers' awareness of the associated factors with cholelithiasis to ensure effective management for SCD patients.
